# Life history strategy and romantic satisfaction in patients’ behavior

**DOI:** 10.3389/fpsyg.2024.1346597

**Published:** 2024-08-20

**Authors:** Cristina Ene, Vlad Burtaverde, Peter Karl Jonason, Felix Brehar, Viorel Pruna

**Affiliations:** ^1^Faculty of Psychology and Educational Sciences, University of Bucharest, Bucharest, Romania; ^2^School of Psychology, University of Padua, Padua, Veneto, Italy; ^3^Institute of Psychology, Cardinal Stefan Wyszynski University, Warsaw, Masovian, Poland; ^4^Department of Neurosurgery, Carol Davila University of Medicine and Pharmacy, Bucharest, Romania; ^5^Department of Neurosurgery, Bagdasar-Arseni Clinical Emergency Hospital, Bucharest, Romania

**Keywords:** high-*K* fitness strategy, patient activation, pain catastrophizing, romantic satisfaction, somatic effort

## Abstract

According to evolutionary psychologists, an individual—consciously or not—who allocates resources for somatic effort focuses on homeostasis and the protection of themselves and others. During health crises, patients must choose between mobilizing their remaining resources to either recover or accepting the disease as inevitable. When patients choose to be proactive in terms of protecting their health, are conscientious, and compliant in the recovery process, a high level of patient activation is achieved. Therefore, we examined (*N* = 252) whether the patients’ *K* fitness strategies are predictors for engagement in patient activation-type behavior. In addition, we tested the mediating effect of pain catastrophizing and the moderating effect of romantic satisfaction. We found that people with a medical diagnosis, who were in a romantic relationship, and had high-*K* fitness were much more likely to be activated patients. Moreover, pain catastrophizing mediated the relationship between high-*K* fitness strategy and patient activation, while romantic satisfaction moderated this relationship, amplifying its intensity. The findings highlight the importance of identifying patients’ psychological resources (e.g., high-*K* strategy, romantic satisfaction, or pain perception) to keep them engaged in the health recovery process.

## Introduction

The field of health psychology frequently explores the efficiency of intervention programs, the rate of patient recovery, patient involvement in the recovery process (Hibbard et al., 2007; Greene and Hibbard, 2012; Lee et al., 2022), and understanding the psychological factors that shape health-relevant behaviors (Martino et al., 2019; Ene et al., 2021). Evolutionary psychologists postulate that people are unconsciously motivated to protect themselves from health risk factors (e.g., disgust reaction to potential contamination and pregnancy sickness; Del Giudice, 2014), which is a manifestation of the human survival instinct (Figueredo et al., 2006; Tybur et al., 2012; Hill et al., 2016). Furthermore, these theories suggest that, given a favorable environment conducive to organism development, certain survival and adaptation strategies (High-*K* strategy) are developed. Individuals who align with this type of life history are characterized by attributes such as self-control, high aspirations, and risk avoidance. They tend to lead longer lives and have fewer offspring, on whom they prefer to focus their efforts and resources, being more inclined to undertake long-term endeavors (Dunkel Schetter, 2011). These strategies are presented on a continuum between two extremes: r strategies (unpredictable, risky environments, abuse, and hostility) and *K* strategies (predictable, prosperous environments, support, and emotional warmth).

However, research has traditionally focused on the general tendency of people to react involuntarily by withdrawing from or rejecting risk factors (Waynforth, 2012; Del Giudice, 2014) rather than on the health recovery process of those with medical diagnoses. In the present study, we investigate the adaptation strategies and somatic efforts of individuals who already have a medical diagnosis, depending on their life history. Therefore, we aim to explore the relationship between high-*K* fitness strategy and patient activation among those diagnosed with medical conditions. This includes examining the mediating role of pain catastrophizing and the moderating role of satisfaction in the couple’s relationship as explanatory mechanisms of this relationship.

### Slow life/*K*-selected strategy and patient activation

According to evolutionary psychology, an individual—consciously or not—allocates resources toward somatic effort (e.g., growing a larger body) or reproductive effort (e.g., pursuing mates or investing in offspring; Figueredo et al., 2006; Giosan, 2006). The concept of fitness refers to the ability and potential of individuals to achieve reproductive success, their general adaptability to the environment, and their capacity to survive as long as possible (Giosan et al., 2018). Evolutionary psychologists show that, depending on the high-*K* fitness strategy, an organism’s life is shaped by several factors, including distinct mortality threats because the somatic effort is inherently limited (Figueredo et al., 2006; Hill et al., 2016). Therefore, organisms often face important trade-offs in distributing these resources among several competing life-sustaining components—growth, maintenance, and reproduction—at any given moment in time (Hill et al., 2016).

For individuals to reach a high level of fitness and avoid unfavorable strategies, it is necessary to adapt their strategies to the environment with its finite resources and make compromises in allocating energy to solve certain tasks relevant to fitness (Figueredo et al., 2006; Giosan, 2006). Life history theory postulates that under the influence of threatening factors unconsciously associated with mortality, people tend to allocate more motivational resources to survival than reproduction (Hill et al., 2016). People with a high-*K* fitness strategy are more oriented toward somatic effort and are long-term oriented (i.e., better health and increased lifespan; Figueredo et al., 2006). Therefore, once such individuals learn their health has deteriorated, they may be unconsciously motivated to protect themselves.

Activated patients in disease recovery are those who can manage their symptoms or difficulties, engage in activities that maintain optimal functioning, and are involved in treatment and diagnosis choices (Hibbard et al., 2004). Patient activation includes trusting in one’s own skills and knowledge, which helps manage their health and healthcare (Hibbard et al., 2004). There are favorable effects that patient activation can have on improving patients’ lives and on the physician–patient relationship (Hibbard et al., 2004, 2015; Feys et al., 2016). Few studies to date have focused on individual predictors of patients in disease management, such as personality, patient–physician relationship, resilience, self-efficacy, or race (Alexander et al., 2014; Crijns et al., 2019; Ene et al., 2021). However, to the best of our knowledge, we can affirm that greater patient activation is supported by a secure environment, trusting relationships, good social skills, conscientiousness, emotional stability, and a willingness to cooperate (Alexander et al., 2014; Crijns et al., 2019; Ene et al., 2021). Such aspects can further be associated with the high-*K* fitness strategy (Del Giudice et al., 2015), as somatic effort drives us to protect ourselves from threats, infections, or microbes. Therefore, the more patient activation, the slower the decline in health should be (Hibbard et al., 2015), thereby decreasing mortality risk.

By adopting an evolutionary lens to understand health behaviors, we contend that people who feel secure will be more motivated to make efforts that facilitate their survival. Growing up in an environment with satisfied needs (Ene et al., 2021), which help to develop self-esteem and a sense of security (e.g., resources allocated in somatic and parental efforts specify for slow life), will promote healthy and self-protective behaviors (Hill et al., 2016). In addition, individuals with a high-*K* fitness strategy, by nature, have a stronger self-preservation instinct, higher self-esteem, have easier access to good healthcare, and get involved more often in activities that improve their health or lifestyle (Buss, 2008; Giosan et al., 2018). These are all resources that individuals have easier access to, increasing their chances of going through an easier recovery, the prognosis to be more favorable, and the motivation for health to be almost always higher. Therefore, we expect a high-*K* fitness strategy to predict patients’ activation with a medical diagnosis and become more involved in the recovery process.

People with a high-*K* fitness strategy might lack important qualities as mating partners, leading to low chances of reproductive success, inappropriate adaptation to the environment, and low potential in the mating market (Buss, 2008). Those with a high-*K* fitness strategy who perceive threats as manageable (e.g., colds or flu) might favor increased somatic effort. However, serious or lifespan-shortening diseases, over which they feel little control (e.g., due to generally poor immune competence, chronic illness, or living in an environment rife with virulent pathogens), may accelerate their life history strategies (Hill et al., 2016). It appears that patients who come from disadvantageous early life conditions tend to exhibit elevated levels of pain catastrophizing, heightened perceived sensitivity to pain, and increased levels of pain-related fear (Simon et al., 2022), consequently finding themselves in unfortunate health situations, feeling trapped by fear, anxiety, or a sense of helplessness regarding their ability to improve their condition.

### Pain catastrophizing

Pain catastrophizing is defined as a negative or exaggerated interpretation and orientation toward painful stimuli, often associated with health impairment, perceived disability, pain behavior, fear of movement, and depression (Sullivan et al., 1995; Suso-Ribera et al., 2017). Previous studies have shown that the burden of aging and functionality-limiting illness may be strongly associated with the adoption of a high-*r* strategy (McNamara et al., 1999; Hill et al., 2016). Growing up in an environment with socioeconomic disadvantages (e.g., low housing quality, high crowding, toxic exposure, and low social climate) reduces the likelihood that children will face adverse physical and psychosocial conditions, which can decrease emotional regulation, stress control, and cognitive functions (Simon et al., 2022). Environmental unpredictability, poor early life functioning, and stressful life events have also been associated with pain sensitivity, establishing memories, and associations for future adults (Waller et al., 2020). Therefore, individuals who were raised in such environmental and familial conditions develop a heightened state of alertness regarding pain and illness over time, feeling uncertain about how they will manage and whether they will find sufficient resources to cope or fully recover.

Given that all these risk factors are characteristic of a high-r fitness strategy, we expect that these patients will exhibit a higher perceived vulnerability to illness and catastrophizing if health risks are moderate or severe and if they perceive high levels of unpredictability and harshness. Individuals who are ill with a high-r fitness strategy may report high levels of pain catastrophizing because they might adopt emergency survival strategies, where danger is imminent and resources are dwindling. In such contexts, they allocate fewer resources to somatic efforts and healthcare, focusing instead on mating efforts and achieving reproductive success more quickly before the disease progresses, and of course, being much more accustomed to taking risks in general (Hill et al., 2016). Therefore, we expect that a high-K strategy has an indirect effect on patient activation through pain catastrophizing.

### Romantic satisfaction

Survival and reproduction are fundamental evolutionary challenges, and humans have developed numerous strategies to maximize both (Salkicevic et al., 2014). These strategies encompass various mechanisms to achieve mating and somatic success. Previous studies indicate that high-*K* strategy indicators are significant predictors of couple satisfaction (Olderbak and Figueredo, 2010; Salkicevic et al., 2014). The potential moderating role of romantic satisfaction in the relationship between high-*K* fitness strategy and patient activation accurately reflects the effect of reproductive motivation and fulfilled reproductive success, which contributes to survival.

We know that individuals with a high-K fitness strategy tend to be oriented toward long-term relationships and have a greater need for stability in relationships (Figueredo et al., 2006; Del Giudice et al., 2015). Therefore, in contexts where people have safety, resources, and support, once they have reproduced, they can better connect to the goal of caring for and investing in their offspring (Del Giudice et al., 2015). Consequently, they find fulfillment and emotional resources in their children and family system, not merely a biological success motivated strictly by instinct.

There is evidence indicating that a slow strategy is frequently associated with long-term romantic orientation, active investment, and emotional involvement in the family system and child-rearing (Figueredo et al., 2006; Del Giudice et al., 2015). Therefore, a stable and satisfying relationship generally makes people feel that they have resources, support, and safety, thus creating a stronger motivation for the patient to focus more on somatic effort rather than reproductive effort. Patients thus have a higher tendency to take care of their health, recover, and regain functionality when all these conditions are met. Therefore, we expect that high relationship satisfaction serves as a reinforcing mechanism for individuals with increased fitness, aiding their physical recovery, enhancing their will to fight for survival, and making them active participants in protecting their health.

To sum up, considering that almost no research has investigated how life history strategies relate to health behavior, we aimed to examine whether individuals characterized by slow life history strategies are “good” patients who prioritize and value their health. Given the complexity of these relationships, we hypothesized that individuals with slow life history/high-*K* strategies, who also have a good romantic relationship, would be even more involved in patient activation behavior. In addition, we expect that patients with slow life/high-K strategies are more prone to catastrophizing pain, an effect that we anticipate will positively influence their mobilization and contribute to a high level of patient activation.

## Method

### Participants and procedure

We used G*Power to estimate the necessary sample size for significant effect sizes. The minimum required sample size was 197 participants for effect size (*r*) of 0.25, with an alpha set at 0.95 and the statistical power set at 0.95. For this study, 252 participants (M_age_ = 38.80, *SD* = 12.20, 67.9% women, 32.1% men) with an age range between 19 and 97 years were voluntarily recruited from several online groups based on the requirement that they have a medical diagnosis and are involved in a committed relationship. Participants who did not meet these conditions were excluded from the study. Participants reported relationship length in months (*M* = 155, *SD* = 128), with a minimum of 4 months and a maximum of 624 months (approximately 53 years).

The reported diagnoses were coded using the ICD-10-CM tabular list of diseases and injuries, and their frequency was reported as follows: 30.6% reported endocrine, nutritional, and metabolic diseases (E00-E89); 17.5% reported diseases of the musculoskeletal system and connective tissue (M00-M99); 11.1% reported diseases of the digestive system (K00-K95); 9.9% reported diseases of the genitourinary system (N00-N95); 7.1% reported diseases of the respiratory system (J00-J99); 6.0% reported neoplasms (C00-D49); 4.8% reported diseases of the skin and subcutaneous tissue (L00-L99); 4.4% reported diseases of the circulatory system (I00-I99); 4.0% reported diseases of the nervous system (G00-G99); 2.0% reported certain infectious and parasitic diseases (A00-B99); 2.0% reported diseases of the blood and blood-forming organs and certain disorders involving the immune mechanism (D50-D89); and 0.8% reported diseases of the ear and mastoid process (H60-H95). All participants were informed about the research objectives and provided consent to participate in the study. Data used in this research can be accessed at https://osf.io/uvxzm/.

### Measures

Patient activation was assessed using the Patient Activation Measure (Hibbard et al., 2004). Participants indicated how much they identified with the items (1 = not at all, 5 = very much) with statements such as *“How confident are you that you can follow through on medical treatments you need to do at home?.”* The scale includes 22 items, and the items were summed to form a composite score (*α* = 0.92).

The high-K fitness strategy was assessed using the High-*K* Strategy Scale (Giosan, 2006), a measure of the high-K independent fitness criterion. Participants indicated how much they agreed with the items (1 = strongly disagree, 5 = strongly agree) with statements such as *“I am in good physical shape.”* The scale consists of 26 items, and all items were summed to form a composite score (*α* = 0.85).

Pain catastrophizing was assessed with the Pain Catastrophizing Scale (Sullivan et al., 1995). Participants indicated how frequently they experienced pain symptoms (1 = almost never, 5 = almost always) with statements such as *“I cannot seem to keep it out of my mind”* or *“I feel I cannot go on.”* The scale consists of 13 items, and all items were summed to form a composite score (*α* = 0.90).

Romantic satisfaction was assessed with the Relationship Assessment Scale (Hendrick, 1988). Participants indicated how satisfied they were with their romantic relationship (0 = very little/not at all satisfied, 4 = very much/very satisfied) with statements such as *“In general, how satisfied are you with your relationship?.”* The scale consists of seven items, and all items were summed to form a composite score (*α* = 0.90).

### Data analysis

The analyses were conducted using SPSS (v25.0) and the MedMod package for R and Jamovi. Pearson’s correlations were used to analyze the relationships between the variables. Hierarchical linear regression was employed to investigate the predictive power of the high-K strategy within romantic relationships on patient activation. To test the mediation effect of pain catastrophizing and the moderation role of romantic satisfaction, we used the Medmod package for R and Jamovi.

## Results

The results presented in Table 1 show descriptive statistics and all correlations among the study variables. People with slow life history strategies reported high patient activation, low pain catastrophizing, and high romantic satisfaction, and people with high patient activation reported low pain catastrophizing. To test the predictive power of the high-*K* fitness strategies on patient activation, we conducted a hierarchical linear regression (Table 2). In Step 1, we introduced high-*K* strategies that predicted 11% of the variance of patient activation (*R*^2^ = 0.11; *F* [1, 218] = 28.1, *p* < 0.001). In Step 2, we included romantic satisfaction and pain catastrophizing. These variables added a 4% increment in the prediction of patient activation (Δ*R*^2^ = 0.04; *F* [3, 216] = 12.8, *p* < 0.001). Overall, the final model with high-*K* fitness strategies, romantic satisfaction, and pain catastrophizing explained 15% of the variance in patient activation.

**Table 1:** Bivariate correlations and descriptive statistics for all the variables of the study.

Variables	1	2	3	4	*M* (*SD*)
1. Patient activation	–				72.30 (11.40)
2. High-*K* fitness strategy	0.38**	–			86.40 (14.70)
3. Romantic satisfaction	0.21**	0.33**	–		29.01 (5.93)
4. Pain catastrophizing	−0.26**	−0.28**	−0.13*	–	22.21 (15.10)

*p < 0.05 and **p < 0.01.

**Table 2:** Hierarchical linear regression on the predictive power of high-*K* fitness strategy, pain catastrophizing, and romantic relationship for patient activation.

Step	Independent variable	Patient activation			Lower BCI	Upper PCI
		*β* (*SE*)	*R*^2^	∆*R*^2^		
1	High*-K* Fitness Strategy	0.05**	0.11**		0.16	0.37
2	Pain catastrophizing	0.05*	0.15**	0.04**	−0.24	−0.03
	Romantic Relationship	0.13			−0.08	0.46

*p < 0.05 and **p < 0.01.

**Figure 1:**
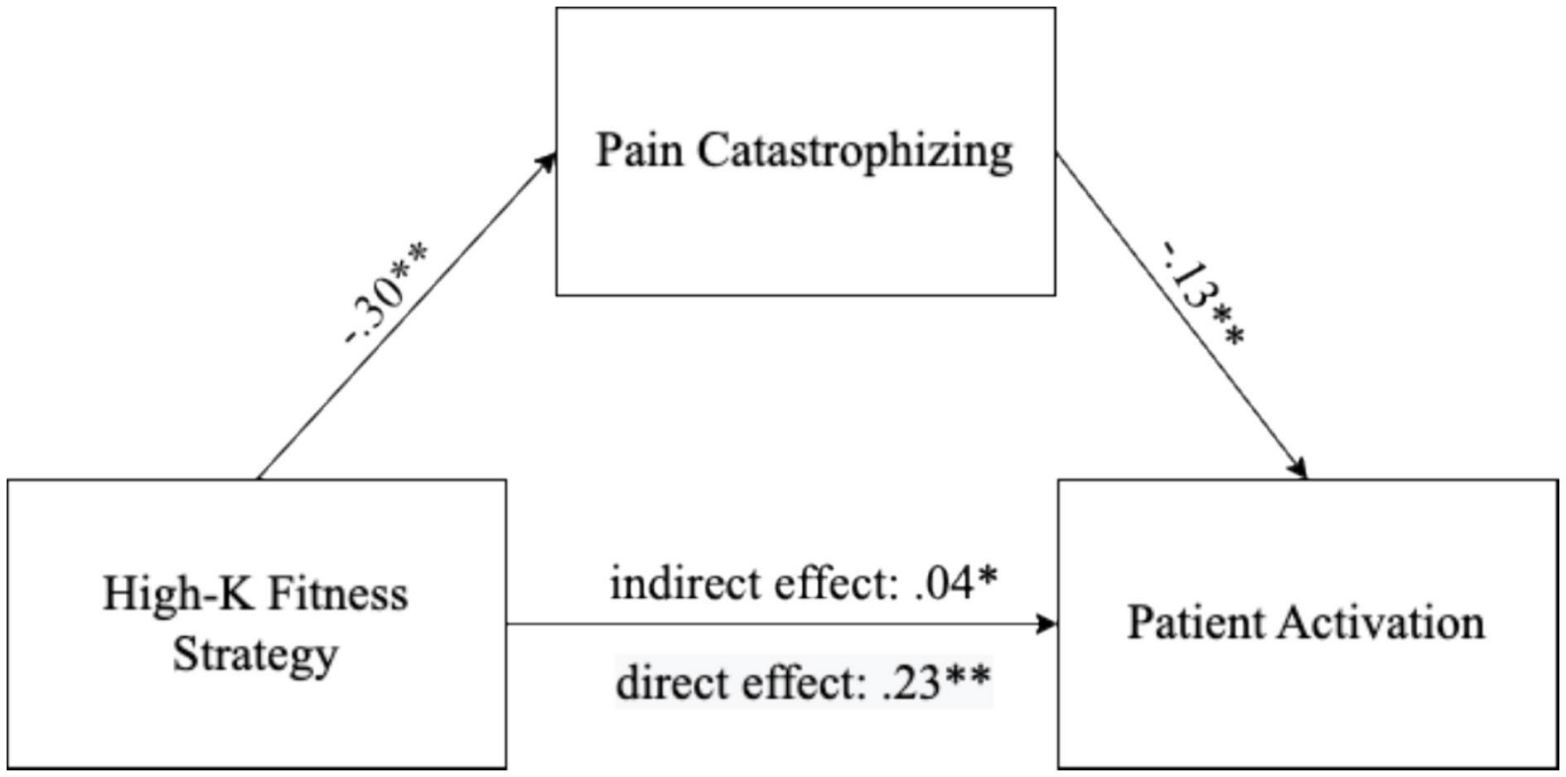
Mediator effect of pain catastrophizing on the relationship between high-*K* fitness strategy and patient activation. *p < 0.05 and **p < 0.01.

We performed an analysis to test whether pain catastrophizing mediated the relationship between the high-*K* fitness strategies and patient activation (Figure 1). People with high-*K* fitness strategies reported low pain catastrophizing (*β* = −0.30, *z* = −4.52, *p* < 0.001). People with high pain catastrophizing reported low patient activation (*β* = −0.13, *z* = −2.59, *p* < 0.05). In addition, people with high-*K* fitness strategies reported high patient activation (*β* = 0.23, *z* = 4.42, *p* < 0.001). Moreover, slow life history strategies led to high patient activation through pain catastrophizing, suggesting partial mediation (indirect effect = *β* = 0.04, *z* = 2.25, *p* < 0.05; direct effect = *β* = 0.23, *z* = 4.42, *p* < 0.001).

**Figure 2:**
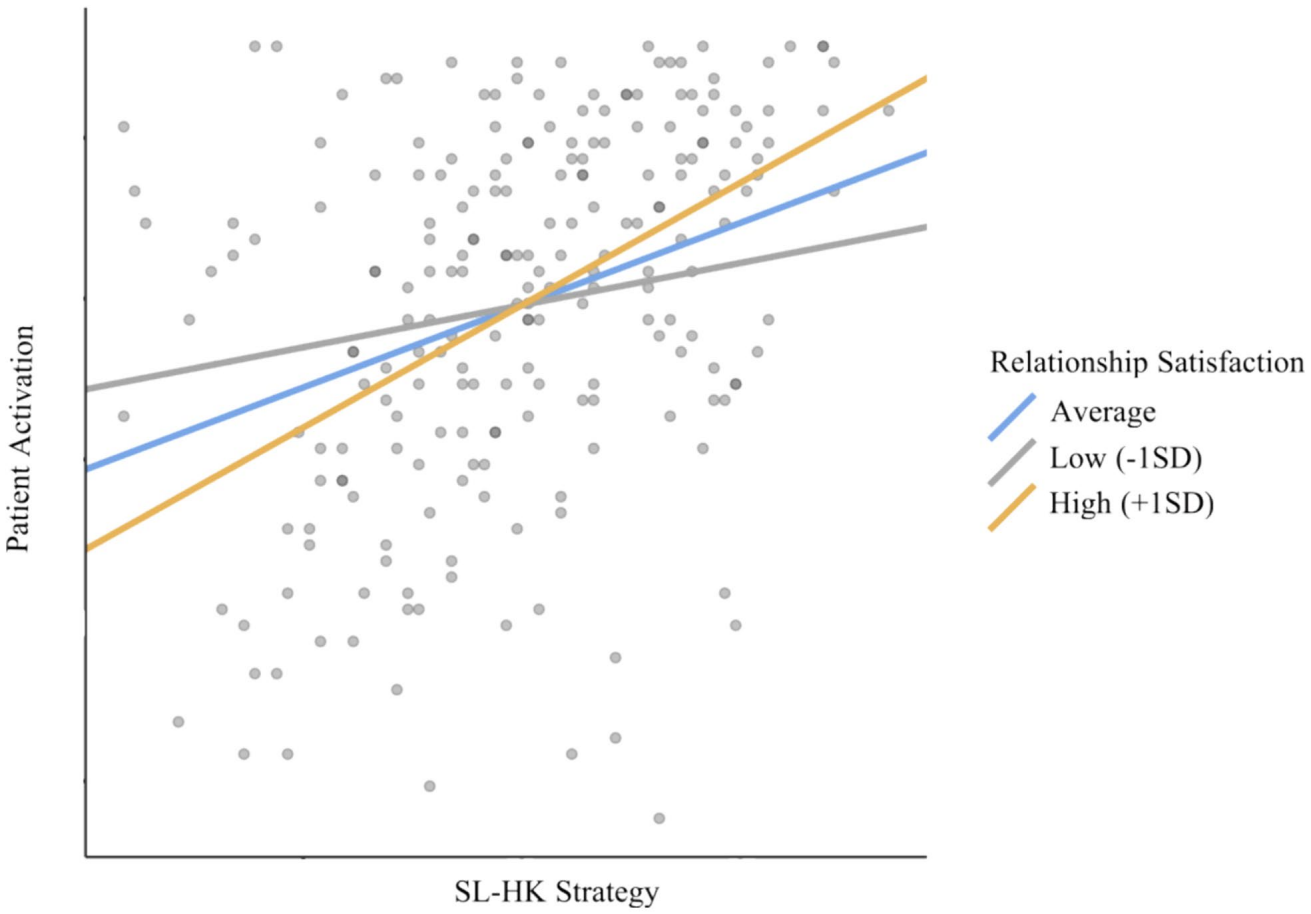
Simple slope plot for the moderating role of romantic satisfaction on the relationship between high-*K* fitness strategy and patient activation.

We performed a moderation analysis (Table 3; Figure 2), and our results showed that patients with high-*K* fitness strategies and higher romantic satisfaction were found to be more activated in recovery and treatment (with 1 unit increase in SD, *B* = 0.33, *z* = 4.75, *p* < 0.01). We also found that for patients with high-*K* fitness strategies and lower romantic satisfaction, even if they reported less activated behavior in recovery and treatment process, the moderation effect has decreased, becoming almost insignificant (with 1 unit increase in SD, *B* = 0.07, *z* = 1.93, and *p* = 0.053).

**Table 3:** Moderator effect of romantic satisfaction on the relationship between high-*K* fitness strategy and patient activation.

Variable	*B* (*SE*)	*z*
High-*K* fitness strategy	0.23 (0.05)	4.54**
Patient activation	0.32 (0.13)	2.57**
High-*K* fitness strategy *×* Patient activation	0.02 (0.01)	2.15*

*p < 0.05 and **p < 0.01.

## Discussion

Considering that almost no research investigated how life history strategies relate to health behavior, we aimed to investigate whether people characterized by slow life history strategies are “good” patients as they should prioritize and value their health. As there are hardly bivariate relationships, we hypothesized that pain catastrophizing mediates the relationships between life history strategies and patient activation, with relationship satisfaction playing a moderating role. We showed that people with slow life history strategies are “good” patients, as they are involved in patient activation behavior. In addition, pain catastrophizing partially mediated the relationship between slow life history strategies and patient activation. Romantic satisfaction moderated the relationship between high-*K* fitness strategies and patient activation, making it more intense.

Individuals who have a slow life history strategy were more likely to have more behaviors characteristic of the patient’s activation. Patients with a high-*K* strategy are people who grow in a secure environment and can have a higher mate value, more resources, and fewer risks. They can be more oriented toward somatic effort and are more interested and preoccupied with their health or direct threats to their survival (Figueredo et al., 2006; Giosan, 2006). Therefore, they can afford to manage a medical diagnosis with more care and involvement. As a result, they can easily become patients who are interested in or compliant with the doctor’s advice, follow the treatment, and do their research about the diagnosis. Therefore, we are referring to a life context in which patients know that if they follow these instructions and are conscientious and compliant with treatment, their chances of healing or survival are higher than if they acted otherwise.

Pain catastrophizing mediates the relationship, with patients using a high-K strategy tending to have more adaptive coping mechanisms related to the disease. These patients try to focus as little as possible on the intensity of the pain, thereby avoiding amplifying their perception of symptoms. This mediation effect is consistent with the argument that patients with a high-*K* strategy are concerned about their health and motivated to protect it as best as possible, not wanting to take additional risks with their health. Therefore, patients with emotional and financial resources, as well as those who actively experience a sense of security, have more coping strategies and become more easily concerned and conscientious in managing their illness.

On the other hand, we can recall previous studies that show us that people who grow up in fast-paced life environments tend to perceive themselves as unattractive, with few qualities (Dillon et al., 2013), and resign about their health with a high assumption of risks and, most likely, a strong orientation toward reproductive efforts (Giosan et al., 2018). People with such undesirable characteristics from the fast life environment were less likely to engage in preventive behavior and lower sense of self-efficacy in disease management (Nowak et al., 2020). Individuals from unpredictable environments are typically characterized by high risk-taking tendencies, an individualistic perspective on the world, and a strong orientation toward reproductive efforts, possibly even perceived as “insensitive” or dominant (Figueredo et al., 2006; Del Giudice et al., 2012). These are adaptive mechanisms to the reality in which they developed, helping them survive in a dangerous and unpredictable environment, and they tend to retain these mechanisms almost invariably into adulthood. However, this does not exclude the existence of emotions in the psychological system of patients from fast environments; rather, it is likely that they resort to adaptive and defensive mechanisms they have learned to be useful when faced with a challenge or danger (e.g., denial, dissociation, rationalization, and displacement; Colovic et al., 2016; Gori et al., 2023). They tend to use all these creative strategies to inhibit anxiety or fear that might otherwise overwhelm them, and sometimes, people may even underreport such emotional experiences.

Moreover, high-*r* individuals end up catastrophizing the pain because living in an unpredictable and poor environment they perceive illness and pain as a greater threat and evaluate risks as more difficult to manage (e.g., lower chances of recovery, higher long-term risks of mortality, or worsening of pain; Hill et al., 2016). Therefore, pain catastrophizing may act as an adaptive mechanism that signals to an individual the threats that are present in unstable environments, helping her/him avoid them. Therefore, they end up investing their limited resources and advantages in an objective that has more chances to be achieved, such as mating and reproductive efforts.

The importance of mating and romantic life is well known (Buss, 2008) and shows how strong people are guided by the “survival instinct.” The results showed that individuals with a slow life history strategy, once they have a satisfactory romantic relationship, will be even more motivated to allocate resources and importance to physical recovery. People know that they need energy and personal resources to fulfill their goals, in general (e.g., to raise their children). Therefore, when patients have a good romantic relationship, they notice that there are chances of success in terms of mating, and they manage to mobilize themselves to effectively manage the somatic aspect as well, as being active in the healing process.

These results have several practical implications. This information could be used in psychotherapy with clients who have health problems and end up being demotivated or frustrated because they have lost their physical functioning, or the process of recovery seems too arduous or protracted. Given that the motivation for survival and reproduction are powerful unconscious mechanisms (Buss, 2008), it is most likely that, under a suitable approach for each client, psychotherapists can help them recreate their goals and follow actions regarding their own lives.

### Limitations and conclusion

However, the study has several limitations. First, our study was cross-sectional, which does not allow us to reveal causal conclusions regarding the realities between the study variables. Second, given that high-*K* fitness strategies are related to higher reproductive success (Figueredo et al., 2006; Giosan et al., 2018), it would have been helpful to check whether participants had children, the time spent with the kids, the age of having become a parent to be able to observe if there are any differences at the level of motivation for somatic efforts, and the specific *K* orientation. Third, the implications of the participants’ diagnoses were not investigated. Each diagnosis can have a different level of severity, with different chances of recovery, which can lead to different attitudes of patients regarding the level of involvement they are willing to allocate to recovery. For future studies, we recommend that researchers investigate the impact of illness severity on the desire to allocate resources to somatic or reproductive efforts. In addition, the indirect effect within the mediation was quite weak, which is why the results should be interpreted with skepticism, and further replication studies will be necessary.

We aimed to bring together the patients’ activation in the recovery considering the aspects that concern life history theory and evolutionary psychology, thus having a more complex perspective on the life of individuals with a medical diagnosis. In this study, we can highlight again the importance of life history as a general framework for orientation in the way the individual chooses to mobilize his resources according to a cost–benefit analysis for success that he makes regarding his resources. We can also extract from this study the importance of a satisfactory couple relationship in the process of recovery from the disease, and this has a motivating role.

## Data availability statement

Publicly available datasets were analyzed in this study. This data can be found here: https://osf.io/shmdg/?view_only=79d4471ef94e4d9c8581a59bc7102525.

## Ethics statement

The studies involving humans were approved by the Ethics Committee of the University of Bucharest. The studies were conducted in accordance with the local legislation and institutional requirements. The participants provided their written informed consent to participate in this study.

## Author contributions

CE: Writing – original draft, Methodology, Investigation, Conceptualization. VB: Writing – review & editing, Validation, Formal analysis, Data curation, Conceptualization. PJ: Writing – review & editing, Supervision, Conceptualization. FB: Conceptualization, Data curation, Formal analysis, Methodology, Resources, Writing – review & editing. VP: Conceptualization, Data curation, Methodology, Resources, Writing – review & editing.
